# The SME tool supporting employers of small- and medium-sized enterprises during the return to work of employees on long-term sick leave: study protocol for a randomized controlled trial and for a process evaluation

**DOI:** 10.1186/s13063-024-08383-4

**Published:** 2024-08-16

**Authors:** Donna C. E. Beerda, Michiel A. Greidanus, Angelique E. de Rijk, Astrid de Wind, Sietske J. Tamminga, Frederieke G. Schaafsma

**Affiliations:** 1 Department of Public and Occupational Health, Amsterdam UMC Location University of Amsterdam, Meibergdreef 9, Amsterdam, the Netherlands; 2 Amsterdam Public Health Research Institute, Societal Participation & Health, Amsterdam, the Netherlands; 3 Department of Social Medicine, Faculty of Health, Medicine and Life Sciences, Research Institute Primary Care and Public Health (CAPHRI), Maastricht University, Duboisdomein 30, Maastricht, the Netherlands

**Keywords:** Return to work, Web-based intervention, Small- and medium-sized enterprises, Randomized controlled trial, Employer support

## Abstract

**Background:**

Returning to work after long-term sick leave can be challenging, particularly in small- and medium-sized enterprises (SMEs) where support may be limited. Recognizing the responsibilities and challenges of SME employers, a web-based intervention (hereafter the SME tool) has been developed. The SME tool aims to enhance the employer’s intention and ability to support the sick-listed employee. Based on the Self-Determination Theory, it is hypothesized that this intention is enhanced by intervening in the employer’s autonomy, competences, and relatedness targeted at, e.g., communication with sick-listed employee, involvement of other stakeholders, and practical support. This is achieved by means of providing templates, communication videos, and information on legislation. This article describes the design of an effect and process evaluation of the SME tool.

**Methods:**

A randomized controlled trial (RCT) with a 6-month follow-up will be conducted with a parallel-group design with two arms: an intervention group and a control group. Sick-listed employees (≤ 8 weeks) of SMEs (≤ 250 employees) at risk of long-term sick leave and their employers will be recruited and randomly allocated as a dyad (1:1). Employers randomized to the intervention group receive unlimited access to the SME tool, while those in the control group will receive care as usual. The primary outcome is the satisfaction of the employee with the return to work (RTW) support provided by their employer. Secondary outcomes include social support, work performance, and quality of work life at the employee level and self-efficacy in providing RTW support at the employer level. Outcomes will be assessed using questionnaires at baseline and 1, 3, and 6 months of follow-up. Process evaluation measures include, e.g., recruitment and use of and perceived usefulness of the SME tool. Additionally, semi-structured interviews with employers, employees, and occupational physicians will explore the interpretation of the RCT results and strategies for the national implementation of the SME tool.

**Discussion:**

The SME tool is hypothesized to be valuable in addition to usual care helping employers to effectively support the RTW of their long-term sick-listed employees, by improving the employers’ intention and ability to support.

**Trial registration:**

ClinicalTrials.gov, NCT06330415. Registered on February 14, 2024.

**Supplementary Information:**

The online version contains supplementary material available at 10.1186/s13063-024-08383-4.

## Introduction

Employees working in small- and medium-sized enterprises (SMEs) represent the majority of the workforce and, as such, contribute significantly to the global economy [1]. To illustrate, SMEs comprised 99% of all enterprises in the European Union and employed 66% of all employees in 2020 [1]. Although lower rates of sick leave occur in SMEs, compared to larger organizations, they are also more vulnerable to the consequences of such absence [2–6].

Compared to larger organizations, owners of smaller enterprises face limitations in financial and practical resources, and they often lack the expertise to support employees’ return to work (RTW) [6]. Resources and expertise include human resources, formal procedures, extended income payments, and access to specialized expertise in managing workplace accommodations or employee support [6–8]. Employers of smaller enterprises have expressed uncertainty regarding their engagement with involved parties and regarding legislation during the RTW [9]. They often have many different responsibilities besides addressing RTW matters, certainly after extended periods of workforce absence [4, 6, 10]. Although employers of SMEs generally maintain close relationships with their employees, time constraints and conflicting interests can reduce the provided RTW support and as a consequence the employee’s satisfaction with the received support [8, 11, 12].

To address the challenges faced by SME employers in supporting the RTW of employees, a web-based intervention (hereafter the SME tool) targeted at employers of SMEs has been developed [13]. As SMEs have been identified as hard to reach, web-based interventions are promising for employers of SMEs to provide convenient, accessible, and cost-effective resources [14]. The SME tool aims to improve the intention and ability of SME employers to provide appropriate support during sick leave and RTW, thereby enhancing the satisfaction of long-term sick-listed employees with the received support from their employer.

The SME tool is developed with the use of the Intervention Mapping (IM) approach, which follows a systematic six-step approach to intervention development [15], and focuses on changing the behavior of the target group, that is in our case, of employers. The desired behavioral change of employers in providing support is outlined in a logic model of change (see Additional file 1) and based on the Self-Determination Theory [16]. This behavioral change theory is chosen because it aligns with the specific characteristics of employers in SMEs. In order to improve the intention to express the behavior of supporting RTW, that is the intrinsic motivation of employers, three behavioral determinants are targeted. Firstly, the component *autonomy* relates to aligning actions with company norms and values, emphasizing independence and flexibility in decision-making, which is crucial in the dynamic nature of SMEs. This is for example reflected in wanting flexibility in when which stakeholder is involved. Secondly, the component *competence* involves the skills and knowledge necessary, for example, for providing practical RTW support. Lastly, the component *relatedness* aligns with the family-like structure often found in SMEs, for example fostering good communication [4, 8].

A randomized controlled trial (RCT) will allow an assessment of the contribution of the SME tool beyond usual care. Research examining employers and employees together within one study design is scarce, but the protocol presented in this study is based on and aligned with previous research and recommendations [17, 18]. By addressing the needs of SME employers, the SME tool could fill an important gap in usual care. Interventions targeting SME employers have, so far, not frequently been developed and evaluated, thus emphasizing the importance for a comprehensive process evaluation to be incorporated as well [19].

This paper describes the design of an RCT that aims to evaluate the effectiveness of the SME tool on the satisfaction of the employee with the RTW support provided by their employer, in comparison to a control group of employer–employee dyads receiving care as usual. The aim of the process evaluation is to clarify the results regarding the effectiveness, to improve the tool if needed, and to explore potential strategies for the national implementation of the SME tool, in case the intervention proves to be effective.

## Methods

### Design

**Fig. 1:**
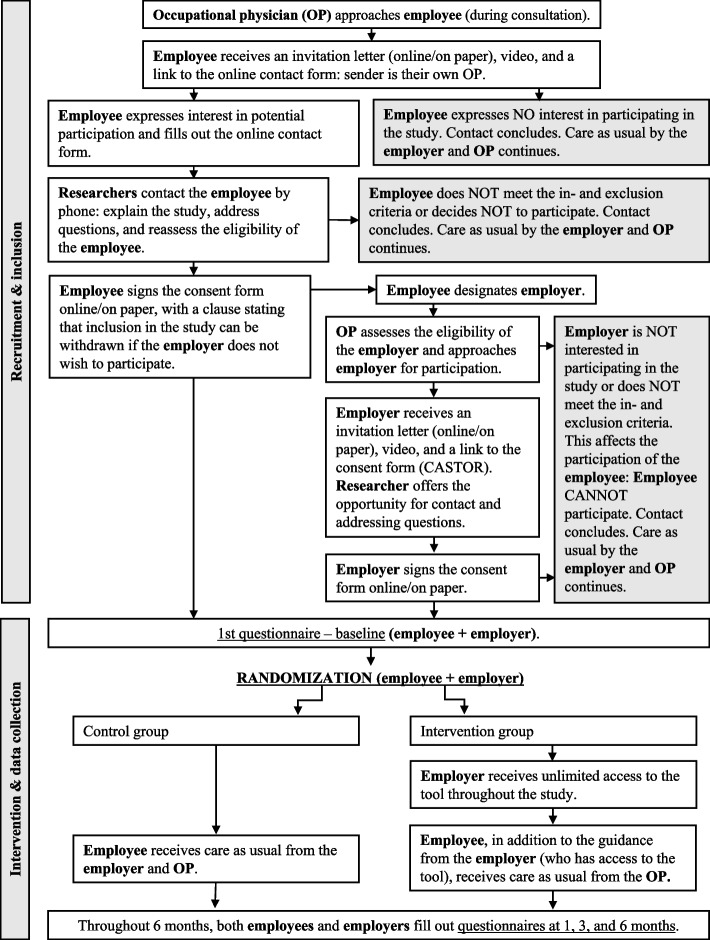
A participant flowchart of the randomized controlled trial

The study will employ a two-arm RCT design with a 6-month follow-up and a process evaluation. The study will be conducted in the Netherlands. For details regarding the Dutch context, we refer to Table 1. Table 2 depicts the timeline from enrolment to measurement timing. The flowchart of the study design is shown in Fig. 1. The study compares employer–employee dyads randomized to the intervention group with employer–employee dyads randomized to the control group. The trial will use a parallel group design with an allocation ratio of 1:1 and follows a superiority framework. The Standard Protocol Items for Clinical Trials (SPIRIT) guidelines were used in the writing of this protocol (see Additional file 2) [20]. The study protocol has been reviewed by the Medical Ethical Review Committee (METC) of the Amsterdam University Medical Center (Amsterdam UMC), the Netherlands (METC2023.0880). The METC has exempted the study protocol from ethical review according to the Dutch Medical Research Involving Human Subjects Act (WMO). In addition to the trial registration (NCT06330415), all items of the World Health Organization Trial Registration Data Set are outlined in Additional file 3.

**Table 1:** Occupational healthcare in the Netherlands

This study is conducted in the Netherlands, where the employer together with the sick-listed employee is responsible for the RTW during the first 2 years of sick leave [21, 22]. Employers are obligated to continue paying the employee’s salary during these first 2 years, though the specific percentage varies based on the collective labor agreement or employment contract [22]. The Gatekeeper Improvement Act outlines the shared responsibility of employers and employees during sick leave and RTW, emphasizing a structured RTW approach [17, 20]. In the Dutch occupational healthcare system the involvement of an occupational physician (OP) is mandatory in case of risk of long-term sick leave. OPs fulfill a consultative role, acting as key intermediaries between employers, employees and the healthcare system. Their expertise lies among others in translating employees’ medical restrictions into work-related constraints or work(place) adaptations that need to be addressed for RTW [22].

**Table 2:**
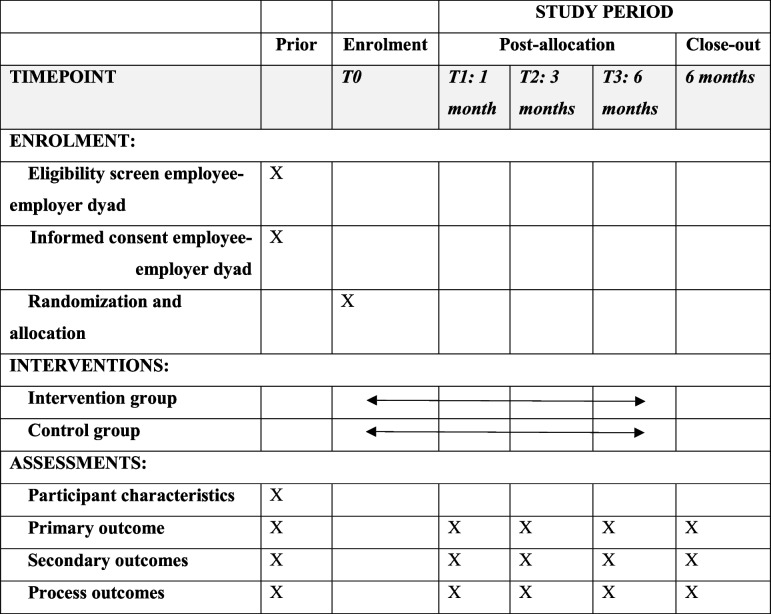
A schedule of enrolment, intervention, and assessments

### Participants

We aim to include 202 employer–employee dyads, thus a total of 404 participants. Both employees and employers will receive a voucher of 20 euros after participating in the study, i.e., when all questionnaires are completed. Employees at study enrolment should:

- Be of working age (18–65 years);
- Have a fixed or temporary employment contract at a SME (≤ 250 employees), with a minimum of 6 months remaining in their contract;
- Be currently sick-listed or partially sick-listed (≤ 8 weeks), with a risk of long-term absenteeism, based on the likelihood of the duration of sick leave exceeding 2 months as assessed by their occupational physician (OP, in plural OPs);
- Be able to understand and read Dutch sufficiently, in order to complete the questionnaires;
- Not have a colleague who is already participating in the study.

Subsequently, the employer of the participating employee will be included. At the time of study entry, the employer should be:

- An employer, supervisor, (case)manager, or HR manager of a SME (≤ 250 employees). In this study, the employer is appointed by the employee and refers to the person who is in direct contact with the employee, and thus the person who offers the RTW support to the employee;
- Able to understand and read Dutch sufficiently, in order to use the SME tool and complete the questionnaires.

### Recruitment

#### Involvement of OPs

Employees will be recruited via approximately 40 OPs. Upon the employee’s consent, the OP will invite the employee’s employer, so that dyads are formed, i.e., employer–employee dyads [18]. We anticipate the involvement of 10 occupational health services, along with multiple freelance OPs, aiming to enlist a total of 40 OPs. We aim to achieve a diversity in the size of occupational health services and the sectors they deliver their service to, with an emphasis on those servicing SMEs. In approaching OPs for the involvement in our study, we will host an informative meeting and use a video to explain the aim and methodology of the study. The executing researcher of the study (DB) provides the OPs with a detailed information package. Continuous communication will be maintained with OPs throughout the study, preceding the inclusion of the dyads and following randomization. OPs are free to recruit additional participants. The OPs will assist in the process but do not handle consent directly. The responsibility for recruiting participants and obtaining informed consent lies with the executing researcher (DB). The executing researcher (DB) and research assistant—under supervision of the principal investigator (FS)—will identify potential recruits and obtain informed consent.

#### Recruitment of employees

Employees will be recruited via OPs in the Netherlands from May 2024. These OPs will approach potential eligible employees during their usual sick leave consultation hours, with an invitation letter, information sheet, consent form, and return envelope. The employees will be asked to return this form to the executing researcher of the study (DB) if they are interested and willing to participate. The above steps can also be carried out digitally, according to the employees’ preferences. Thereafter, researcher DB will contact the employee by phone to explain the study, to check the employee’s eligibility according to the inclusion criteria, and to give the employee the opportunity to ask questions. If an employee is eligible and willing to participate, he or she can sign informed consent (see Additional file 4) and subsequently appoint his or her employer.

#### Recruitment of employers

Subsequently, the OP assesses the eligibility of the employer and will invite eligible employers via telephone or e-mail to participate. A digital informed consent form will be sent to the employer (see Additional file 4). The executing researcher (DB) offers the opportunity for contact and addressing questions. In case the employer is not interested in participation, the employee is unable to participate in the study, as only employer–employee dyads can participate. After signing the form, the dyads will be assigned a unique number in chronological order of inclusion, starting at 001. After inclusion, participants will be asked to complete the baseline questionnaire digitally and the dyads will thereafter be randomized to the intervention or the control group.

## Sample size calculation

The sample size calculation for this RCT is based on the primary outcome measure: the linear rate of change in the employees’ perceived satisfaction with their employer’s RTW support with respect to the satisfaction at baseline, over a period of 6 months after the beginning of the trial. Based on a prior study involving 3470 employees with various conditions, the expected mean score for the question on satisfaction at baseline is considered to be 2.64 (range 1–5) with a standard deviation of 1.05 [23]. Assuming a 20% improvement after 6 months in the intervention group and no relative improvement in the control group, it is anticipated that the intervention group will achieve a mean score of 3.17 resulting in a linear change rate of 0.53 over a period of 6 months. We estimate the effect of the SME tool on our primary outcome using a linear mixed model with a random intercept and slope for each OP to account for clustering due to OPs recruiting employer–employee dyads. In this model, we estimate the linear effect of the time, and the interaction between time and treatment on the primary outcome using the data collected at 3 and 6 months follow-up. The models are also adjusted for the satisfaction outcome value at the baseline. Assuming an intracluster correlation coefficient of 0.1, both the intervention and control arms need 80 employees to achieve 80% statistical power for detecting a significant difference in the change rate of 0.088 per month of the outcome over time between the intervention and control group at the significance level $\alpha =0.05$ [24]. In total, we plan to recruit 202 dyads, which equals 404 participants, to allow for an attrition rate of 20%. The sample size calculation was performed using nQuery [24].

## Randomization

Employer–employee dyads are randomly assigned to either the intervention or control group in a 1:1 ratio with random block sizes. Neither the participants nor the researchers will be blinded. Randomization is performed in Castor EDC [25] using variable block randomization to reduce the predictability of the random sequence. The executing researcher (DB) will conduct the randomization upon receiving the informed consent forms. In instances where multiple participants require randomization at the same time, another researcher will conduct the randomization. Allocation concealment will be ensured in Castor EDC, with the release of the randomization code only occurring after the completion of all baseline measurements. The allocation to the intervention or control group will be definitive, with no possibility of reversal.

### The intervention

The SME tool comprises of an open-access website targeting the employer of the sick-listed employee. The website provides practical resources, including templates, communication videos and information on legislation, possible workplace accommodations, and tailored tips on how to support employees with most common reasons of sick leave. In line with legislation [22] and based on previous experience with an online employer tool for supporting employees with cancer [26], information is tailored to different phases of sick leave: (1) the time of reporting ill, (2) short-term sick leave (≤ 6 weeks), (3) long-term sick leave (> 6 weeks), (4) RTW, and (5) follow-up care. A comprehensive overview of the intervention’s development, objectives, and elements will be available in a publication elsewhere [13]. In brief, the SME tool has been developed using the IM approach, incorporating insights from stakeholders: (walk-through) interviews with employers (*N* = 14) and focus groups with employees (*N* = 12) and OPs (*N* = 12) [27]. The SME tool aims to improve the intention and ability of SME employers to provide appropriate support during sick leave and RTW, thereby enhancing the satisfaction of long-term sick-listed employees with the received support from their employer. The desired change of employer behavior, aimed at providing RTW support, is outlined in a logical model of change (see Additional file 1) using the Self-Determination Theory [15, 16, 26]. This theory resonates with distinct characteristics inherent to SME employers, and its three key components are connected to the targeted behavior changes. To begin, the relatedness component aligns with the familial structure often found in SMEs, such as fostering good communication [8]. Subsequently, the autonomy component is associated with aligning actions with company norms and values, emphasizing independence and flexibility in decision-making—a crucial aspect in the dynamic SME environment. For instance, engaging the right stakeholders and experiencing freedom in doing so. Finally, the competence component aligns with the essential skills and knowledge for tasks such as providing practical RTW support.

## Intervention group

After the inclusion of the employee–employer dyad, the SME tool is provided to the employers randomized to the intervention group. The executing researcher (DB) will provide these employers with an e-mail containing their unique website’s URL and details of the study. Employers are asked to use the SME tool during the 6 months follow-up. They will receive an e-mail after 1 and 3 months reminding them to use it, although its usage is not mandated. There are no criteria for discontinuing the allocated intervention.

## Control group

The URL of the SME tool will remain undisclosed to employers in the control group, ensuring that employers and employees will receive care as usual from their OP. The study will be conducted in the Netherlands. For details regarding the Dutch context and thus the usual care for the control group, we refer to Table 1. During the course of the study, online search engines will not be able to trace the SME tool to prevent possible contamination from the intervention group to the control group. All participants are permitted to receive concomitant care or interventions.

### Data collection

Self-reported questionnaires will be distributed digitally to employees and employers using the electronic data capture system Castor EDM [35], prior to randomization (baseline; T0), and after 1 (T1), 3 (T2), and 6 months of follow-up (T3). E-mail and phone reminders will be sent after 1 and 2 weeks, respectively. All data is securely saved in Castor EDC [35], processed in SPSS 25.0 [28], and stored on the secure sponsor’s surfer. The executing researcher (DB), principal investigator (FG), and project leaders (MG and ST) will be given access to the final data sets. No prior adverse events have been reported during digital employer interventions [17], and we do not anticipate any. However, should any adverse events occur, we will report them through the sponsor’s portal at the Amsterdam UMC.

## Participant characteristics and effect measures

### Employees

At T0, the questionnaire contains questions about the personal and work-related characteristics of the employee (e.g., age, gender, reason for sick leave, educational level, work status, type of contract, type of work). Measures for effect evaluation will be assessed as change from baseline at each time point (i.e., T0, T1, T2, and T3), except for one time to event: *total number of sick leave days*. Table 3 depicts an overview of all outcome variables.

**Table 3:** An overview of primary, secondary, and process outcome variables

Level	Construct	Measurement instrument; # items	Example item	Response categories	Score calculation
Primary outcome variable					
Employee	Satisfaction with the RTW support from employer	Questionnaire; Self-developed; 1 item	How satisfied are you with the sick leave and return to work guidance you have received from your employer since the sick leave/over the past month/3 months?	1) Very dissatisfied, 2) dissatisfied, 3) neutral, 4) satisfied, and 5) very satisfied	N/A
Secondary outcome variable					
Employee	Social support from employer	Questionnaire; Self-developed; 8 items	How important is it that your employer provides emotional support during sick leave and RTW? (importance); how much emotional support have you perceived form your employer? (amount of support)	1) Very unimportant, 2) unimportant, 3) neutral, 4) important, and 5) very important; 1) Very few, 2) few, 3) neutral, 4) much, and 5) very much	“Importance score” multiplied with “amount of support score” leads to “weighted score”, with higher scores indicating more social support from employer
	Total number of sick leave days	Questionnaire; Self-developed; 8 items	Have you worked in the past month/3 months? This can include both activities in your regular job as well as in adapted work	Yes or no options and open-ended responses to express current employment status, for example date input (e.g., return to work since:../../….)	The average number of working days per week divided by the contracted work days
	Work performance	Questionnaire; Maastricht Instrument for Sustainable Employability (MAISE), subscale 2A ‘work performance’; 6 items	I have the knowledge to perform my job well	1) Completely disagree, 2) disagree, 3) neutral, 4) agree, and 5) completely agree	Mean score, with higher scores indicating better work performance
	Quality of Working Life	Questionnaire; Quality of Working Life Questionnaire for cancer survivors (QWLQ-CS), subscale 2 **“**understanding and recognition in the organization”; 5 items	My supervisor understands my health situation and possible complaints	1) Completely disagree, 2) disagree, 3) somewhat disagree, 4) somewhat agree, 5) agree, and 6) completely agree	Mean score, with higher scores indicating higher quality of working life
Employer	Self-efficacy	Questionnaire; Empowerment Questionnaire, subscale “the competence scale”; 3 items	Supporting an employee who has reported ill is a significant responsibility for me	1) Completely disagree, 2) disagree, 3) somewhat disagree, 4) somewhat agree, 5) agree, and 6) completely agree	Mean score, with higher scores indicating more self-efficacy
	Satisfaction with the resumption of work of the respective employee	Questionnaire; Self-developed; 1 item	How satisfied are you with the work resumption process of your employee since the sick leave/over the past month/3 months?	1) Very dissatisfied, 2) dissatisfied, 3) neutral, 4) satisfied, and 5) very satisfied	N/A
Process evaluation					
Employer	Recruitment	Questionnaire and logbook of in- and exclusion of (potential) participants; Self-developed; 1 item	What is your reason for not participating?	Open-ended	N/A
	Use of the SME tool	Questionnaire and tracking software (Piwik PRO); Self-developed 6 items	How often have you visited the SME tool in the past three months?	Open-ended, multiple-choice options, and website usage	N/A
	Perceived usefulness of the SME tool	Questionnaire; Self-developed; 9 items	Would you recommend the tool to other employers when guiding an employee who has reported sick?	Open-ended and multiple-choice options	N/A
	Components of the logic model of change	Questionnaire; Self-developed; 3 items	I have the skills to support an employee on sick leave	1) Completely disagree, 2) disagree, 3) somewhat disagree, 4) somewhat agree, 5) agree, and 6) completely agree	Individual scores represent the overall score of individual components, with higher scores indicating higher autonomy, competence, relatedness and ability
	Experiences	Topic list; Self-developed; 5 items	Potential implementation strategies for the national implementation of the SME tool	Open-ended	N/A
Employee	Recruitment	Questionnaire and logbook of in- and exclusion of (potential) participants; Self-developed; 1 item	What is your reason for not participating?	Open-ended	N/A
	Experiences	Topic-list; Self-developed; 5 items	Potential implementation strategies for the national implementation of the SME tool	Open-ended	N/A

*N/A* not applicable

The primary outcome will be the *employee satisfaction with the RTW support from employer*, measured with a 1-item rating: “How satisfied are you with the sick leave and return to work guidance you have received from your employer since your sick leave/over the past month/3 months?” scored on a 5-point Likert scale, from very dissatisfied to very satisfied. The category represents the score, with higher scores indicating higher satisfaction. Satisfaction, in this context, refers to the general attitude towards experiences encountered [23]. Similar inquiries on job satisfaction or satisfaction with specific job aspects are commonly addressed in scientific literature [29, 30].

Secondary outcomes on the level of the employee include:

- Social support: social support is assessed through the four facets of support potentially provided by the employer, i.e.: (1) emotional support, (2) practical support, (3) informational support, and (4) appreciative support [23, 31]. The measurement comprises eight questions regarding the importance and amount of support received. The mean score of the importance and amount of support are analyzed separately to take into account the individual difference between the perceived importance. Multiplying all the “importance scores” with each corresponding “amount of support score” leads to a total “weighted score”. A higher score indicates higher social support from the employer, collectively assessing the overall level of social support provided by the employer [32].
- Total number of sick leave days: the total number of sick leave days involves the assessment of the average number of working days per week over the preceding 4 weeks and the total number of (partial) sick days. This is measured with questions about the current employment status, continuity in the same organization and job position, recent work activities, and details about returning to work if applicable [33]. The total number of sick leave days status is calculated by dividing the average number of working days per week by the contracted work days (T0–T3). Additionally, the total count of (partial) sick days is obtained by measuring the time interval between the initial day of illness and the date of resuming work.
- Work performance: work performance is measured with subscale 2A from the Maastricht Instrument for Sustainable Employability (MAISE) and refers to the long-term capacity of employees to actively participate in and contribute to the workforce [34]. The six statements, only filled in if work activities have been done in the past 4 weeks, are measured on a 5-point Likert scale. The mean score of the total sum is calculated, with higher scores indicating higher work performance. These scales have demonstrated reliability and validity across diverse employee populations [34].
- Quality of working life: quality of working life is measured with subscale 2 from the Quality of Working Life Questionnaire for cancer survivors (QWLQ-CS). This validated questionnaire comprises items designed to capture dimensions of work-related perceptions and is measured on a 6-point Likert scale. Only subscale 2 about “understanding and recognition in the organization” consisting of five items is included, as we only anticipate change in this subscale according to our logic model of change (Additional file 1). The sum represents the overall score, with a higher score corresponding to a higher feeling of understanding and recognition in the organization. The questionnaire’s internal consistency, construct validity, and group-level reproducibility have been established, rendering it suitable as a patient-reported outcome measure for interventions [35, 36]. The questionnaire has also been applied beyond cancer-related contexts, such as in cases of chronic inflammatory bowel disease [37].

### Employers

At T0, the questionnaire contains questions about the characteristics and occupation of the employer (e.g., age, gender, educational level, sector, and size of the organization (i.e., number of employees)). At the level of the employer, the secondary outcome measures that will be assessed are as follows:

- Self-efficacy: self-efficacy refers to the perceived ability of the employer to effectively support employees during sick leave and RTW. This measure is assessed with three items regarding meaningfulness, impact, and skills from the competence scale of the Empowerment Questionnaire [38]. Statements are measured on a 6-point Likert scale. The mean score of the sum is calculated, with higher scores indicating higher self-efficacy [38].
- Satisfaction with the resumption of work of the respective employee: this is measured with one self-developed question: “How satisfied are you with the work resumption process of your employee since the sick leave/over the past month/3 months?” on a 5-point Likert scale. The category represents the overall score, with a higher score indicating higher satisfaction.

## Process evaluation

In addition to the effect outcomes, the following process outcomes will be assessed at the level of the employee/employer, according to the model by Linnan and Steckler [13]:

- Recruitment: recruitment is measured by assessing the amount and reasons for non-participation of dyads and the usefulness of recruitment strategies used by the researchers. This is measured in CASTOR, or on paper via a return envelope, with an open-ended question, filled in by recruited employees who do not wish to participate prior to the trial. Also, a logbook related to recruitment strategies is maintained by the researchers, containing details about what recruitment strategy is used during the trial and the corresponding outcome.
- Use of the SME tool: the use of the SME tool is measured with self-developed questions regarding number of visits, duration of visits, tool availability, and web analytics (T2–T3). Employers are issued personalized links to enhance the tracking of web analytics using Piwik PRO. From this information, we can derive the administered and received dose [17, 39].
- Perceived usefulness of the SME tool: the perceived usefulness of the SME tool is measured with self-developed questions regarding the usefulness of the tool, enhancement of knowledge and skills, and likelihood of recommending the tool to colleagues (T3) [17].
- Components of the logic model of change: according to our logic model of change (see Additional file 1), we expect a change on the level of autonomy, competence, relatedness, and ability (T1–T3). These four are measured in self-developed questions and measured on a 6-point Likert scale. Individual scores represent the overall score of individual components, with higher scores indicating higher autonomy, competence, relatedness, and ability.
- Experiences: the interpretation of the results from the effective measurement and potential implementation strategies for the national implementation of the tool will be explored. One-time interviews will be conducted with 5–10 employees, 5–10 employers, and 5–10 OPs, out of a purposive sample of the RCT participants (after T3). Participants will be selected and invited for voluntary participation in a one-time interview lasting a maximum of 45 min, based on responses from the T3 questionnaire, including factors such as employee satisfaction and perceived utility, aiming for a diverse sample. Topics include (1) the use of the SME tool (employers randomized to the intervention group), (2) the perceived utility of the SME tool (employers randomized to the intervention group and occupational health physicians), (3) perceived support (employees), (4) the implemented implementation strategy for the effectiveness evaluation (all participants), and (5) potential implementation strategies for the national implementation of the SME tool (all participants).

### Statistical analysis

All data will be analyzed according to the intention-to-treat (ITT) principle, as well as the per-protocol (PP) principle [17]. The latter analysis is conducted by tracking the analytics of the website usage through Piwik PRO at least one time if randomized to the intervention group [39]. Due to the nature of the intervention, blinding of participants is not possible. Therefore, the trial is open-labeled. However, data analysis will be performed by a researcher who is blinded to the arms. Primary and secondary outcomes and participants’ characteristics for effect evaluation will be assessed using descriptive statistics. *P*-values ≤ 0.05 will be considered statistically significant. Longitudinal linear mixed models (LMM), including random intercepts and random slopes for each OP, will be used to examine differences between the intervention and control groups for the continuous primary outcome measures as well as all continuous secondary outcome measures: social support from the employer, work performance, quality of working life, self-efficacy, and satisfaction with the resumption of work of the respective employee. Kaplan–Meier survival analysis and Cox regression analysis will be used to compare the time from the first day of sick leave to RTW between the intervention and control groups. In the Kaplan–Meier survival analysis, we will censor patients who dropped out of the study. Covariate adjustment will be performed in the models to account for factors such as gender and age that appear to be prognostic for the outcome measures. We will report reasons for dropout for each randomization group and compare the reasons quantitatively and qualitatively. Dropouts will be included in the analysis by modern imputation methods for missing data. The interviews for the process evaluation will be recorded, transcribed verbatim, and thematically analyzed using MaxQDA [40]. The statistical analyses, including data preparation, will be conducted using SPSS 25.0 and R [28, 41].

## Discussion

Returning to work after long-term sick leave can be challenging, particularly in SMEs where resources and support may be limited. Recognizing the specific responsibilities and challenges SME employers have, the SME tool has been developed to assist in guiding the RTW of employees on long-term sick leave. To determine the effectiveness of the SME tool on the satisfaction of long-term sick-listed employees with the support from their supervisor, an RCT has been designed for effect evaluation and complemented with a process evaluation.

## Methodological considerations

The SME tool is developed in accordance to the needs and expectations of the SME employers, employees, and OPs. Its relevance and potential impact are further enhanced by its foundation in the Self-Determination Theory and its execution according to the IM approach, building upon previous intervention-developing experiences [15–17]. Additionally, the study incorporates both employers and employees, a methodological approach aligning with previous recommendations [17, 18]. Therefore, the range of outcome measures is on various levels, contributing to a comprehensive understanding of the SME tool’s effect. The additional process evaluation adds to this understanding by providing in-depth insight on how the SME tool is experienced, complementing the quantitative findings. Moreover, the primary outcome measure is focused on the employee. Our choice for the primary outcome measure was guided by the logic model of change (see Additional file 1), expecting that improved RTW support from the employer will lead to higher employee satisfaction and, ultimately, sustainable RTW. However, sustainable RTW is also influenced by many other factors beyond the potential intervention’s impact, for example, the severity of the employee’s condition [42, 43] and sustainable RTW is thus not an outcome of this study.

Several limitations should be considered with regard to the study design. Firstly, blinding of the intervention is not feasible, as both employer–employee dyads are aware of group assignment, potentially influencing outcomes [44]. For example, employees in the intervention group might be more positive about their employer due to their access to the SME tool. Similarly, employers assigned to the intervention group may prompt a more favorable self-assessment. Some e-health interventions opt for a placebo e-health intervention to minimize bias in estimating the intervention’s effects [45]. There is considerable variation among placebo e-health interventions, but they typically incorporate existing information that is part of usual care (e.g., a patient information folder that is normally provided by the hospital on paper) [45, 46]. We have chosen not to develop a placebo tool for the control group since there is no placebo alternative available that truly mimics the content of the intervention under assessment. Additionally, introducing a placebo tool for SMEs that lacks substantial information may raise ethical concerns. Besides, the potential awareness among participants in the control group that they are not receiving a genuine intervention could lead to frustration, which could adversely elevate dropout rates [47, 48]. Secondly, instances where employers decline participation despite the employee willingness present an unfortunate constraint, as the study design requires the employer–employee dyads, hampering the external validity. Lastly, we explored alternative study designs involving different clusters and recruitment strategies. One alternative was cluster randomization on the level of the recruiting OPs. However, we decided that the drawbacks of contamination effects outweigh the drawbacks associated with the potential baseline differences in cluster randomization, for example, due to regional differences or age [49].

## Impact of results

The potential results of the proposed RCT evaluating the effectiveness of the SME tool may have significant implications for improving RTW support in SMEs and the use of evidence-based interventions to support employers throughout this process of RTW support. By addressing the unique needs of SMEs, the SME tool could fill an important gap in existing RTW services, offering an easily accessible and potentially effective support for SME employers, ultimately contributing to the employee’s RTW. Although the study is conducted in the Netherlands, its findings are expected to be of international value. SMEs globally encounter similar challenges during long-term sick leave of employees, making the insights applicable internationally [3, 4, 6, 8, 9]. The expected improvement of employers’ intention and ability to support the employee’s RTW could lead to informed decision-making and better implementation of supportive workplace practices for their long-term sick-listed employees.

## Trial status

The study was registered in Clinicaltrials.gov on February 14, 2024 (NCT06330415), at www.clinicaltrials.gov/study/NCT06330415. The current manuscript describes the same protocol as registered in Clinicaltrials.gov (version 1.0, February 14, 2024). Recruitment of participants started in May 2024 and the primary completion is expected in May 2025. The results of the RCT are expected in August 2025.

## Supplementary Information

Additional file 1. Logic model of change

Additional file 2. Completed SPIRIT checklist

Additional file 3. World Health Organization Trial Registration Data Set

Additional file 4. Informed consent employee and employer

## Data Availability

Data sharing will be possible upon reasonable request.

## Abbreviations

SMEs: Small- and medium-sized enterprises
RCT: Randomized controlled trial
RTW: Return to work
SPIRIT: Standard Protocol Items for Clinical Trials
METC: Medical Ethics Review Committee (in Dutch: Medisch Ethische Toetsings Commissie)
OP: Occupational physician (in plural: OPs)
N/a: Not applicable
MAISE: Maastricht Instrument for Sustainable Employability
QWLQ-CS: Quality of Working Life Questionnaire for Cancer Survivors
ITT: Intention-to-treat
PP: Per protocol
LMM: Longitudinal linear mixed models
IM: Intervention mapping
