# Supplementary material for: The SME tool supporting employers of small- and medium-sized enterprises during the return to work of employees on long-term sick leave: study protocol for a randomized controlled trial and for a process evaluation
Source: Trials. 2024 Aug 16;25:541. doi: 10.1186/s13063-024-08383-4 (PMC11328497; doi:10.1186/s13063-024-08383-4)
Supplement: Supplementary file 1 — Additional file 1. Logic model of change [file 13063_2024_8383_MOESM1_ESM.pptx]

## Slide 1
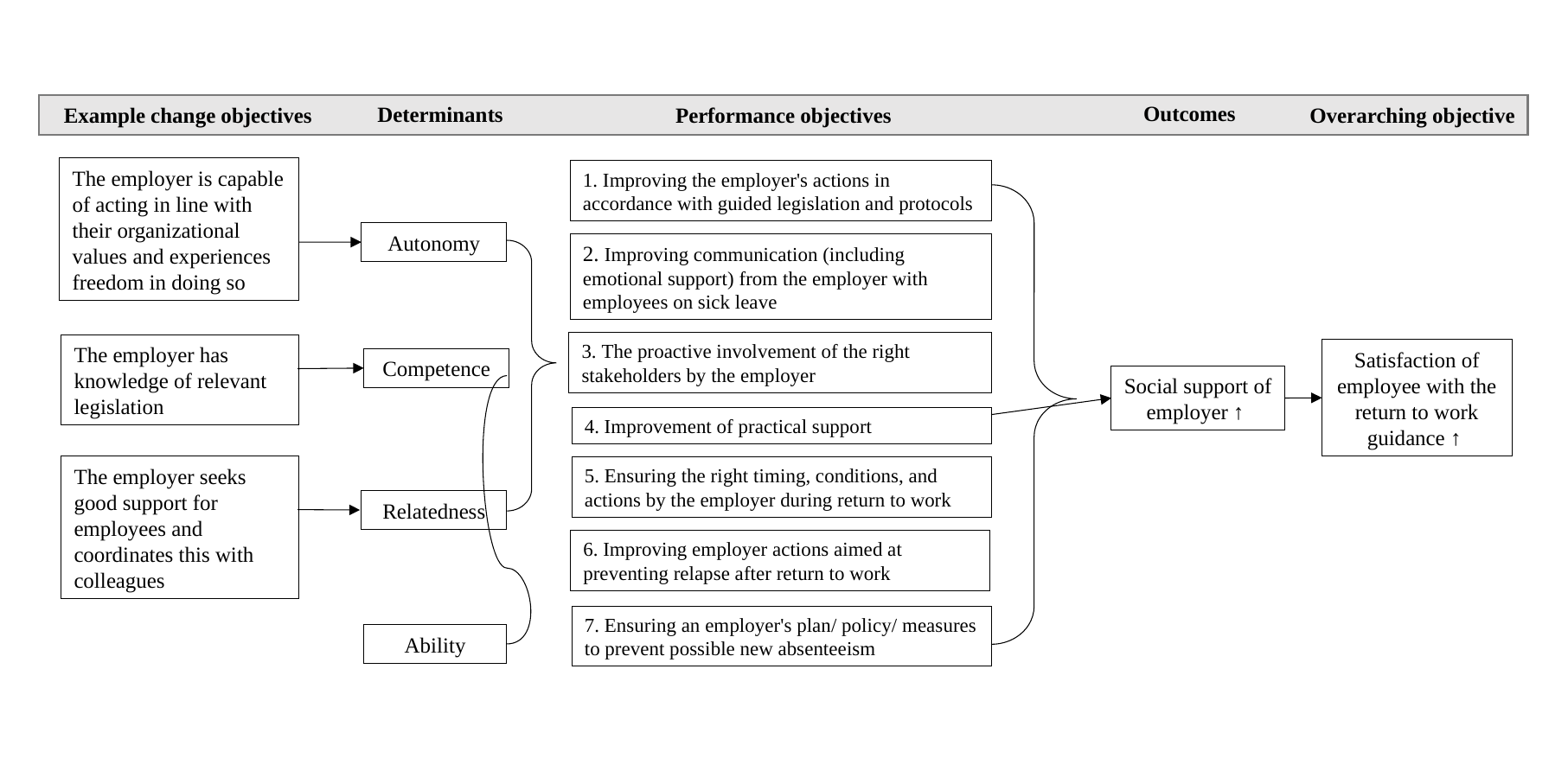

Outcomes
Determinants
Example change objectives
Overarching objective
Performance objectives
The employer is capable of acting in line with their organizational values and experiences freedom in doing so
1. Improving the employer's actions in accordance with guided legislation and protocols
Autonomy
2. Improving communication (including emotional support) from the employer with employees on sick leave
3. The proactive involvement of the right stakeholders by the employer
The employer has knowledge of relevant legislation
Satisfaction of employee with the return to work guidance ↑
Competence
Social support of employer ↑
4. Improvement of practical support
The employer seeks good support for employees and coordinates this with colleagues
5. Ensuring the right timing, conditions, and actions by the employer during return to work
Relatedness
6. Improving employer actions aimed at preventing relapse after return to work
7. Ensuring an employer's plan/ policy/ measures to prevent possible new absenteeism
Ability
