# Supplementary material for: The SME tool supporting employers of small- and medium-sized enterprises during the return to work of employees on long-term sick leave: study protocol for a randomized controlled trial and for a process evaluation
Source: Trials. 2024 Aug 16;25:541. doi: 10.1186/s13063-024-08383-4 (PMC11328497; doi:10.1186/s13063-024-08383-4)
Supplement: Supplementary file 3 — Additional file 3. World Health Organization Trial Registration Data Set [file 13063_2024_8383_MOESM3_ESM.docx]

**Appendix 3**. World Health Organization Trial Registration Data Set

**Table 3.** All items from the World Health Organization Trial Registration Data Set

| Data category | Information^32^ |
| --- | --- |
| Primary registry and trial identifying number | ClinicalTrials.gov NCT06330415 |
| Date of registration in primary registry | 14 February, 2024 |
| Secondary identifying numbers | N/a |
| Source(s) of monetary or material support | ZonMw: The Netherlands Organisation for Health Research and Development |
| Primary sponsor | Academisch Medisch Centrum - Universiteit van Amsterdam (AMC-UvA) |
| Secondary sponsor(s) | N/a |
| Contact for public queries | Donna C. E. Beerda  Amsterdam UMC, 1105 AZ Amsterdam, The Netherlands  E-mail: d.c.e.beerda@amsterdamumc.nl |
| Contact for scientific queries | Donna C. E. Beerda  Amsterdam UMC, 1105 AZ Amsterdam, The Netherlands  E-mail: d.c.e.beerda@amsterdamumc.nl |
| Public title | Effect and Process Evaluation of the SME Tool |
| Scientific title | The SME tool supporting employers of small and medium-sized enterprises during the return to work of employees on long-term sick-leave: study protocol for a randomized controlled trial and process evaluation |
| Countries of recruitment | The Netherlands |
| Health condition(s) or problem(s) studied | Sick-listed SME employees |
| Intervention(s) | Intervention group: The SME tool (unlimited access) |
|  | Control group: Care as usual by the occupational health service |
| Key inclusion and exclusion criteria | Inclusion criteria: Employees at study enrolment should:   - be of working age (18-65 years); - have a fixed or temporary employment contract at a SME (≤ 250 employees), with a minimum of 6 months remaining in their contract; - be currently sick-listed or partially sick-listed (≤8 weeks), with a risk of long-term absenteeism, based on the likelihood of the duration of sickness absence exceeding two months as assessed by their occupational physician; - be able to understand and read Dutch sufficiently, in order to complete the questionnaires. |
|  | Inclusion criteria: At time of study entry, the employer should be:   - an employer, supervisor, (case)manager or HR manager of a SME (≤ 250 employees). In this study, the employer is appointed by the employee and refers to the person who is the direct supervisor, in direct contact with the employee, and thus the person who offers the RTW support to the employee; - able to understand and read Dutch sufficiently, in order to use the intervention and complete the questionnaires. |
|  | Exclusion Criteria:  • Not have a colleague who is already participating in the study. |
| Study type | Interventional |
|  | Allocation: randomized intervention model. Parallel assignment masking: no masking (Open Label). |
|  | Primary purpose: Intervention |
|  | Phase III |
| Date of first enrolment | May 2024 |
| Target sample size | 404 |
| Recruitment status | Not yet recruiting |
| Primary outcome(s) | Satisfaction with the RTW support of employer assessed by the employee (Time Frame: Baseline (T0), 1 month (T1), 3 month (T2) and 6 month (T3) follow-up) |
| Key secondary outcomes | - Social support (Time Frame: Baseline (T0), 1 month (T1), 3 month (T2) and 6 month (T3) follow-up) - Total number of sick-leave days (Time Frame: Baseline (T0), 1 month (T1), 3 month (T2) and 6 month (T3) follow-up) - Work performance (Time Frame: Baseline (T0), 1 month (T1), 3 month (T2) and 6 month (T3) follow-up) - Quality of working Life (Time Frame: Baseline (T0), 1 month (T1), 3 month (T2) and 6 month (T3) follow-up) - Self-efficacy (Time Frame: Baseline (T0), 1 month (T1), 3 month (T2) and 6 month (T3) follow-up) - Satisfaction with the resumption of work of the respective employee (Time Frame: Baseline (T0), 1 month (T1), 3 month (T2) and 6 month (T3) follow-up) - Recruitment [Time Frame: 1 month (T1), 3 month (T2) and 6 month (T3) follow-up] - Use of the SME tool [Time Frame: 3 month (T2) and 6 month (T3) follow-up] - Perceived usefulness of the SME tool [Time Frame: 3 month (T2) and 6 month (T3) follow-up] - Components of the logic model of change [Time Frame: 1 month (T1), 3 month (T2) and 6 month (T3) follow-up] - Experiences [Time Frame: Cross-sectional after 6 months follow-up] |
