# Supplementary material for: The SME tool supporting employers of small- and medium-sized enterprises during the return to work of employees on long-term sick leave: study protocol for a randomized controlled trial and for a process evaluation
Source: Trials. 2024 Aug 16;25:541. doi: 10.1186/s13063-024-08383-4 (PMC11328497; doi:10.1186/s13063-024-08383-4)
Supplement: Supplementary file 4 — Additional file 4. Informed consent employee and employer [file 13063_2024_8383_MOESM4_ESM.docx]

**Appendix 4. Informed consent forms**

**Informed consent form employee – English translation**

**The SME tool**

- I have read the information letter. I also had the opportunity to ask any questions. My questions have been sufficiently answered. I had enough time to decide whether or not to participate.
- I know that participation in this study is voluntary. I also understand that I can decide not to participate or to withdraw from the study at any time. I do not need to provide a reason for this.
- I consent to the collection and use of my data in the manner and for the purposes outlined in the information letter.
- I am aware that all information collected will be treated confidentially. The personal and research data can only be accessed by the research team.
- I consent to my data being retained for 10 years after this study within Amsterdam UMC.
- I consent to my employer being contacted regarding potential participation in this study.
- I understand that I am participating in this study together with my employer. If my employer does not wish to participate in this study, I understand that I cannot participate either. I am aware that the researcher will contact me if my employer does not wish to participate.
- I wish to participate in this study
- I do not wish to participate in this study, for the following reason:
- I am not interested in participating in the study.
- I am already participating in another study (note: this does not hinder participation in our study).
- I do not belong to the target group employee, namely: …………………………………
- I prefer not to answer.
- Other, namely: ………………………………………………………………………………………………….

Name:

Place: Date: _____ / _____ / ______

Signature:

- - I give/do not give permission to be contacted for an additional interview during this study (via e-mail or letter):
- Permission granted
- No permission
  - If participating in the interview, I give permission for the collection and the use of audio recordings:
- Permission granted
- No permission
- If participating in the interview, I give permission to retain the collected audio recording for 10 years after completion of the study:
- Permission granted
- No permission
  - I give/do not give permission to be contacted after completion of this study for a follow-up study (via e-mail or letter):
- Permission granted
- No permission
  - Please indicate what applies:

(multiple answers possible)

- After completion of the study, I would like to receive the study results per e-mail.
- After completion of the study, I would like to receive project newsletters per e-mail (maximum 2x per year).
- I am not interested in receiving the study results or project newsletters of this study.

----------------------------------------------------------------------------------------------------------------

*To be completed by the researcher:*

I certify that I have fully informed this participant about the mentioned study. If any information arises during the study that could affect the participant's consent, I will inform him/her promptly.

Name researcher (or representative):

Signature:

Date: _____ / _____ / ______

----------------------------------------------------------------------------------------------------------------

*The participant will receive a complete information letter along with a copy of the signed informed consent form.*

**Informed consent form employer - English translation**

**The SME tool**

- I have read the information letter. I also had the opportunity to ask any questions. My questions have been sufficiently answered. I had enough time to decide whether or not to participate.
- I know that participation in this study is voluntary. I also understand that I can decide not to participate or to withdraw from the study at any time. I do not need to provide a reason for this.
- I consent to the collection and use of my data in the manner and for the purposes outlined in the information letter.
- I am aware that all information collected will be treated confidentially. The personal and research data can only be accessed by the research team.
- I consent to my data being retained for 10 years after this study within Amsterdam UMC.
- I wish to participate in this study
- I do not wish to participate in this study, for the following reason:
- I am not interested in participating in the study.
- I am already participating in another study (note: this does not hinder participation in our study).
- I do not belong to the target group employer, namely: ………………………………….
- I prefer not to answer.
- Other, namely: ………………………………………………………………………………………………….

Name:

Place: Date: _____ / _____ / ______

Signature:

- - I give/do not give permission to be contacted for an additional interview during this study (via e-mail or letter):
- Permission granted
- No permission
  - If participating in the interview, I give permission for the collection and the use of audio recordings:
- Permission granted
- No permission
- If participating in the interview, I give permission to retain the collected audio recording for 10 years after completion of the study:
- Permission granted
- No permission
  - I give/do not give permission to be contacted after completion of this study for a follow-up study (via e-mail or letter):
- Permission granted
- No permission
  - Please indicate what applies:

(multiple answers possible)

- After completion of the study, I would like to receive the study results per e-mail.
- After completion of the study, I would like to receive project newsletters per e-mail (maximum 2x per year).
- I am not interested in receiving the study results or project newsletters of this study.

----------------------------------------------------------------------------------------------------------------

*To be completed by the researcher:*

I certify that I have fully informed this participant about the mentioned study. If any information arises during the study that could affect the participant's consent, I will inform him/her promptly.

Name researcher (or representative):

Signature:

Date: _____ / _____ / ______

----------------------------------------------------------------------------------------------------------------

*The participant will receive a complete information letter along with a copy of the signed informed consent form.*

**Toestemmingsformulier werknemer – original document in Dutch**

**Onderzoek MKB re-integratie tool**

- Ik heb de informatiebrief gelezen. Ook kon ik vragen stellen. Mijn vragen zijn voldoende beantwoord. Ik had genoeg tijd om te beslissen of ik meedoe.
  - Ik weet dat meedoen vrijwillig is. Ook weet ik dat ik op ieder moment kan beslissen om toch niet mee te doen of te stoppen met het onderzoek. Daarvoor hoef ik geen reden te geven.
  - Ik geef toestemming voor het verzamelen en gebruiken van mijn gegevens op de manier en voor de doelen die in de informatiebrief staan.
  - Ik weet dat alle informatie die wordt verzameld, vertrouwelijk wordt behandeld. De persoons- en onderzoeksgegevens zijn alleen in te zien zijn door het onderzoeksteam.
  - Ik geef toestemming om mijn gegevens nog 10 jaar na dit onderzoek te bewaren binnen het Amsterdam UMC.
  - Ik geef toestemming om contact op te nemen met mijn werkgever voor eventuele deelname aan dit onderzoek.
  - Ik weet dat ik samen met mijn werkgever deelneem aan dit onderzoek. Indien mijn werkgever niet wenst deel te nemen aan dit onderzoek, dan weet ik dat ik ook niet kan deelnemen aan het onderzoek. Ik weet dat de onderzoeker contact met mij opneemt als mijn werkgever niet wil deelnemen.
- Ik wil meedoen aan dit onderzoek.
- Ik wil niet meedoen aan dit onderzoek, om de volgende reden:
- Ik heb geen interesse om deel te nemen aan het onderzoek.
- Ik doe al mee aan een ander onderzoek (let op: dit is voor ons geen belemmering voor deelname).
- Ik behoor niet tot de doelgroep, namelijk: …………………………………………………….
- Dat wil ik liever niet beantwoorden.
- Anders, namelijk: …………………………………………………………………………………………….

Naam:

Plaats: Datum : ______ / ______ / ________

Handtekening:

- - Ik geef wel/geen toestemming om mij tijdens dit onderzoek te benaderen voor een aanvullend interview (via e-mail of brief):
- wel toestemming
- geen toestemming
  - Indien deelname aan het interview, geef ik toestemming voor het verzamelen en gebruiken van geluidsopnames.

☐ wel toestemming

☐ geen toestemming

- Indien deelname aan het interview, geef ik toestemming om de verzamelde geluidsopnames ook na het onderzoek nog 10 jaar te bewaren.
- wel toestemming
- geen toestemming
  - Ik geef wel/geen toestemming om mij na dit onderzoek opnieuw te benaderen voor vervolgonderzoek (via e-mail of brief):
- wel toestemming
- geen toestemming
  - Geef hieronder aan wat van toepassing is:

(meerdere antwoorden mogelijk)

- Na afloop van de studie ontvang ik graag per email de resultaten van het onderzoek.
- Na afloop van de studie ontvang ik graag per email de nieuwsbrieven van het project (maximaal 2x per jaar).
- Ik heb geen behoefte om de resultaten van het onderzoek of de nieuwsbrief te ontvangen.

----------------------------------------------------------------------------------------------------------------

*In te vullen door de onderzoeker:*

Ik verklaar dat ik deze deelnemer volledig heb geïnformeerd over het genoemde onderzoek.

Als er tijdens het onderzoek informatie bekend wordt die de toestemming van de deelnemer zou kunnen beïnvloeden, dan breng ik hem/haar daarvan tijdig op de hoogte.

Naam onderzoeker (of diens vertegenwoordiger):

Handtekening:

Datum: _____ / _____ / ______

----------------------------------------------------------------------------------------------------------------

*De deelnemer krijgt een volledige informatiebrief, samen met een kopie van het getekende toestemmingsformulier.*

**Toestemmingsformulier werkgever – original document in Dutch**

**Onderzoek MKB re-integratie tool**

- Ik heb de informatiebrief gelezen. Ook kon ik vragen stellen. Mijn vragen zijn voldoende beantwoord. Ik had genoeg tijd om te beslissen of ik meedoe.
  - Ik weet dat meedoen vrijwillig is. Ook weet ik dat ik op ieder moment kan beslissen om toch niet mee te doen of te stoppen met het onderzoek. Daarvoor hoef ik geen reden te geven.
  - Ik geef toestemming voor het verzamelen en gebruiken van mijn gegevens op de manier en voor de doelen die in de informatiebrief staan.
  - Ik weet dat alle informatie die wordt verzameld, vertrouwelijk wordt behandeld. De persoons- en onderzoeksgegevens zijn alleen in te zien zijn door het onderzoeksteam.
  - Ik geef toestemming om mijn gegevens nog 10 jaar na dit onderzoek te bewaren binnen het Amsterdam UMC.
- Ik wil meedoen aan dit onderzoek.
- Ik wil niet meedoen aan dit onderzoek, om de volgende reden:
- Ik heb geen interesse om deel te nemen aan het onderzoek.
- Ik doe al mee aan een ander onderzoek (let op: dit is voor ons geen belemmering voor deelname).
- Ik behoor niet tot de doelgroep werkgever, namelijk……………
- Dat wil ik liever niet beantwoorden.
- Anders, namelijk: …………………………………………………………………………………………….

Naam werkgever:

Plaats: Datum : ______ / ______ / ________

Handtekening:

- - Ik geef wel/geen toestemming om mij tijdens dit onderzoek te benaderen voor een aanvullend interview (via e-mail of brief):
  - Indien deelname aan het interview, geef ik toestemming voor het verzamelen en gebruiken van geluidsopnames.

☐ wel toestemming

☐ geen toestemming

- Indien deelname aan het interview, geef ik toestemming om de verzamelde geluidsopnames ook na het onderzoek nog 10 jaar te bewaren.
- wel toestemming
- geen toestemming
  - Ik geef wel/geen toestemming om mij na dit onderzoek opnieuw te benaderen voor vervolgonderzoek (via e-mail of brief):
- wel toestemming
- geen toestemming
  - Geef hieronder aan wat van toepassing is:

(meerdere antwoorden mogelijk)

- Na afloop van de studie ontvang ik graag per email de resultaten van het onderzoek.
- Na afloop van de studie ontvang ik graag per email de nieuwsbrieven van het project (maximaal 2x per jaar).
- Ik heb geen behoefte om de resultaten van het onderzoek of de nieuwsbrief te ontvangen.

----------------------------------------------------------------------------------------------------------------

*In te vullen door de onderzoeker:*

Ik verklaar dat ik deze deelnemer volledig heb geïnformeerd over het genoemde onderzoek.

Als er tijdens het onderzoek informatie bekend wordt die de toestemming van de deelnemer zou kunnen beïnvloeden, dan breng ik hem/haar daarvan tijdig op de hoogte.

Naam onderzoeker (of diens vertegenwoordiger):

Handtekening:

Datum: _____ / _____ / ______

----------------------------------------------------------------------------------------------------------------

*De deelnemer krijgt een volledige informatiebrief, samen met een kopie van het getekende toestemmingsformulier.*
